# The Enteric Fever "Carrier"

**Published:** 1910-12-10

**Authors:** 


### The Enteric Fever "Carrier."

An important addition to the series of Local Government Board Reports on Public Health and Medical Subjects has just been published in the form of a review, by Dr. J. C. G. Ledingham, of current knowledge on the subject of the enteric fever "carrier." The Report,* of which we publish a more detailed summary on pages 314-16, consists of fifteen chapters and a Preface by Dr. Theodore Thomson.

A considerable part of Dr. Ledingham's Report is devoted to the literature of the question of principal importance—that, namely, of the infectivity of carriers. The author gives the history of a large number of occurrences of enteric fever in domestic life, in institutions, and in military populations, in which the source of infection has been traced, more or less convincingly, to a carrier. What share of enteric fever is directly due to the carrier is a problem which has been investigated by several observers and the results of their investigations vary markedly, the proportions recorded by different inquirers ranging between 2.3 per cent. and 32 per cent. of the total amount of fever. As to the extent to which carriers exist, the results of investigations go to show that the proportion of carriers in an ordinary population is about three or four per thousand; but the investigations have not been made on such a scale as to permit of these figures being regarded as a trustworthy indication of the true proportion of carriers in communities generally. Dr. Ledingham cites statistics which show that, so far as present data go, the chronic carrier condition appears to be much more frequent in adult life than in youth, and in the female sex than in the male sex. In the latter connection he considers the association that has been observed between the carrier state and gallstone disease and the apparently greater liability of women to both these conditions.

The Report treats with considerable detail the question of the possibility of effecting a cure of the carrier condition, but so far the results of treatment have not been encouraging. There would, however, seem to be somewhat more hope of effecting a cure of the urinary carrier than of the intestinal carrier. The measures that have been taken with a view to lessening the danger of dissemination of enteric fever by carriers are considered, as well as instructions which it has been suggested should be handed to typhoid convalescents. In that way the public, which is at present apt to regard any professional attention to such disseminating centres as in the nature of aggressive medical bullying to harmless and inoffensive convalescents, is likely to be educated to a knowledge of the pernicious results that may follow untreated cases. There is no doubt that much good has already been accomplished by instructing typhoid carriers in proper methods of disinfection, and, in the opinion of the author, it is on the disinfection of the hands that chief stress must be laid.

* *Reports to the Local Government Board on Public Health and Medical Subjects.* New Series, No. 43. Price 1s.

